# Wyoming Valley Veterinary Medical Association1Reported for the Journal by Dr. J. H. Timberman.

**Published:** 1895-09

**Authors:** 


### WYOMING VALLEY VETERINARY MEDICAL ASSOCIATION.[1]

THE regular monthly meeting was held in Scranton, Friday evening, July 26th, at the rooms of the Y. M. C. A. Members present were Dr. Butterfield, President, Montrose; Dr. Church, Secretary, Luzerne; Drs. Helmer and Sturge, Scranton; and Dr. Timberman, Wilkesbarre. The minutes of the last meeting were read and approved.

This was a very interesting meeting, the members seemed to make up in enthusiasm what they lacked in number. A motion to discontinue monthly meetings and to hold quarterly meetings instead elicited considerable discussion, in which a lively and general interest in the life and success of the Association was clearly brought out, each member present seeming to have a desire to do the best for the prosperity and success of the Society. It was the general opinion, however, that the meetings would be better attended if held quarterly, and it was therefore ordered that the meetings should hereafter be held quarterly instead of monthly. The Secretary was ordered to have postal cards printed in such form as to include notice of meeting and programme, to be sent out at least two weeks prior to meeting, and that the Committee on Essays and Papers appoint at least two essayists, to be notified a month prior to meeting.

Dr. Butterfield reported a case of urethral obstruction in a yearling bull, Dr. Sturge an unusually interesting case of melanosis, and Dr. Timberman a case of ruptured diaphragm due to the animal getting fast in his stall during the night.

Adjourned to meet at Dr. Butterfield's farm, South Montrose, on the last Friday in October, at which the doctor promises to have a clinic. This meeting will probably include an afternoon and evening session.

---

### MISSOURI STATE VETERINARY MEDICAL ASSOCIATION.

[1] Reported for the JOURNAL by Dr. J. H. Timberman.

